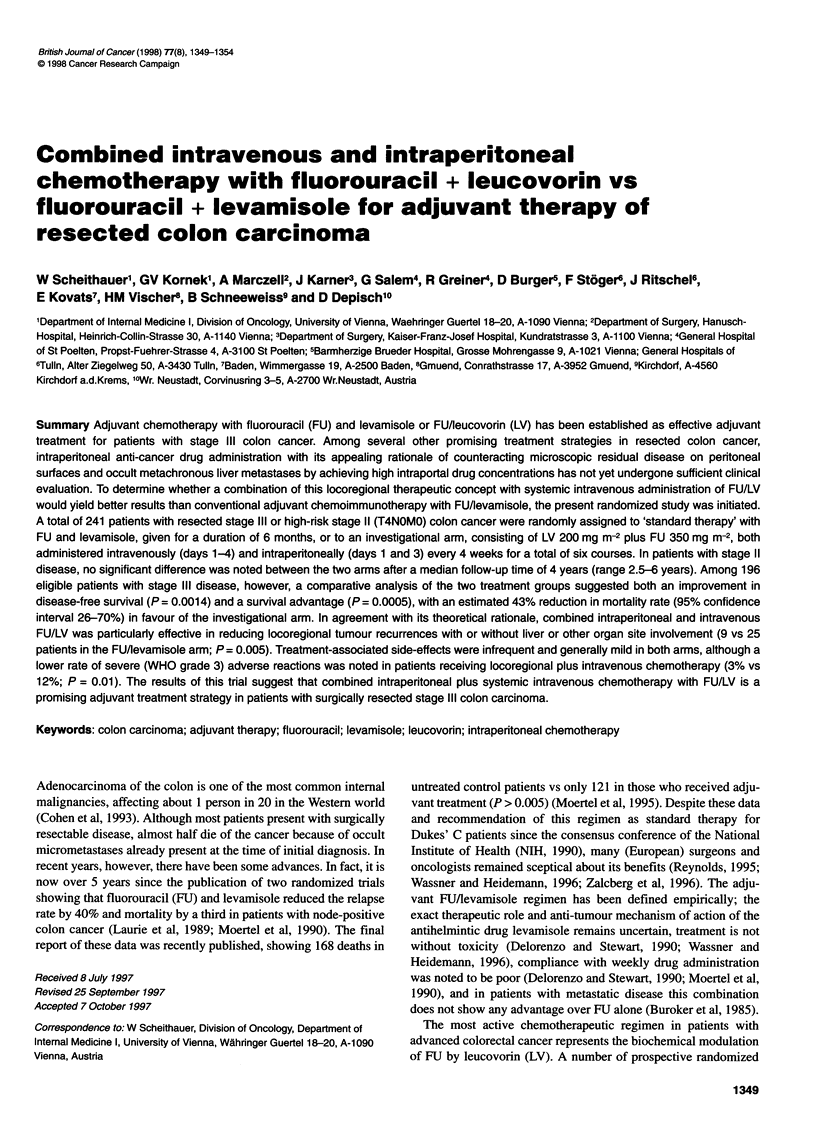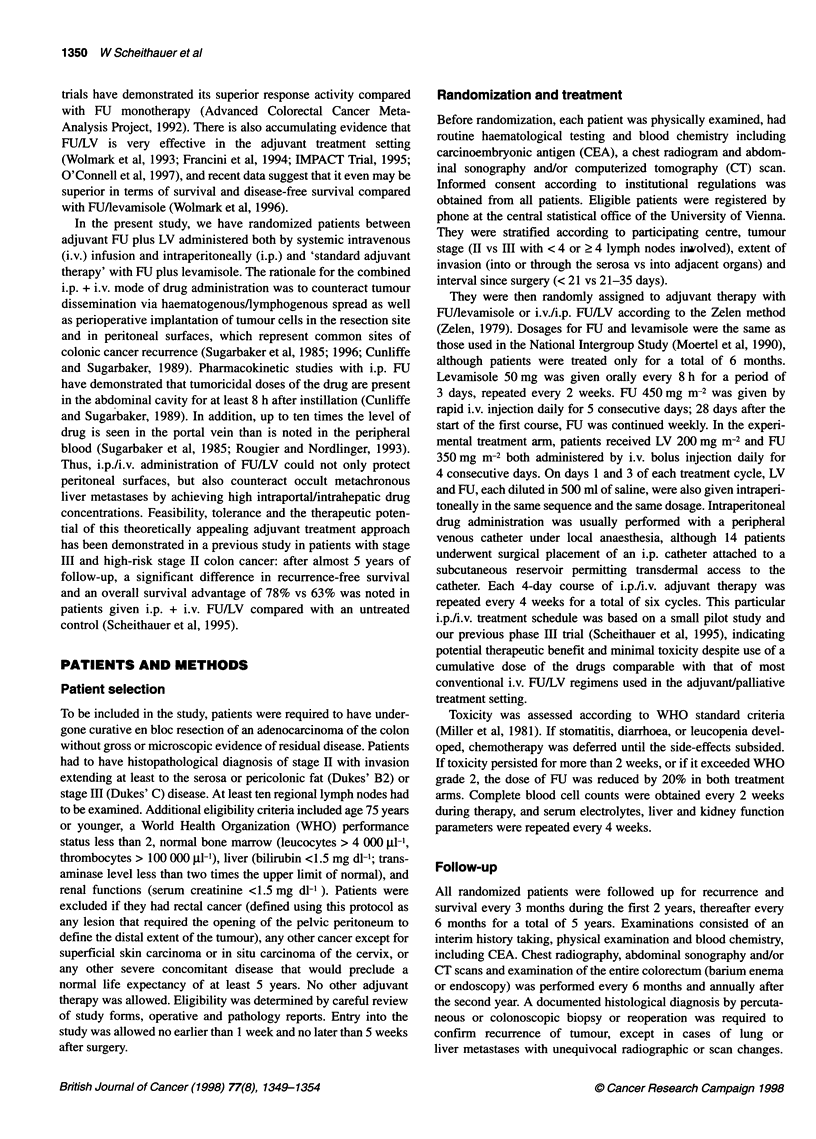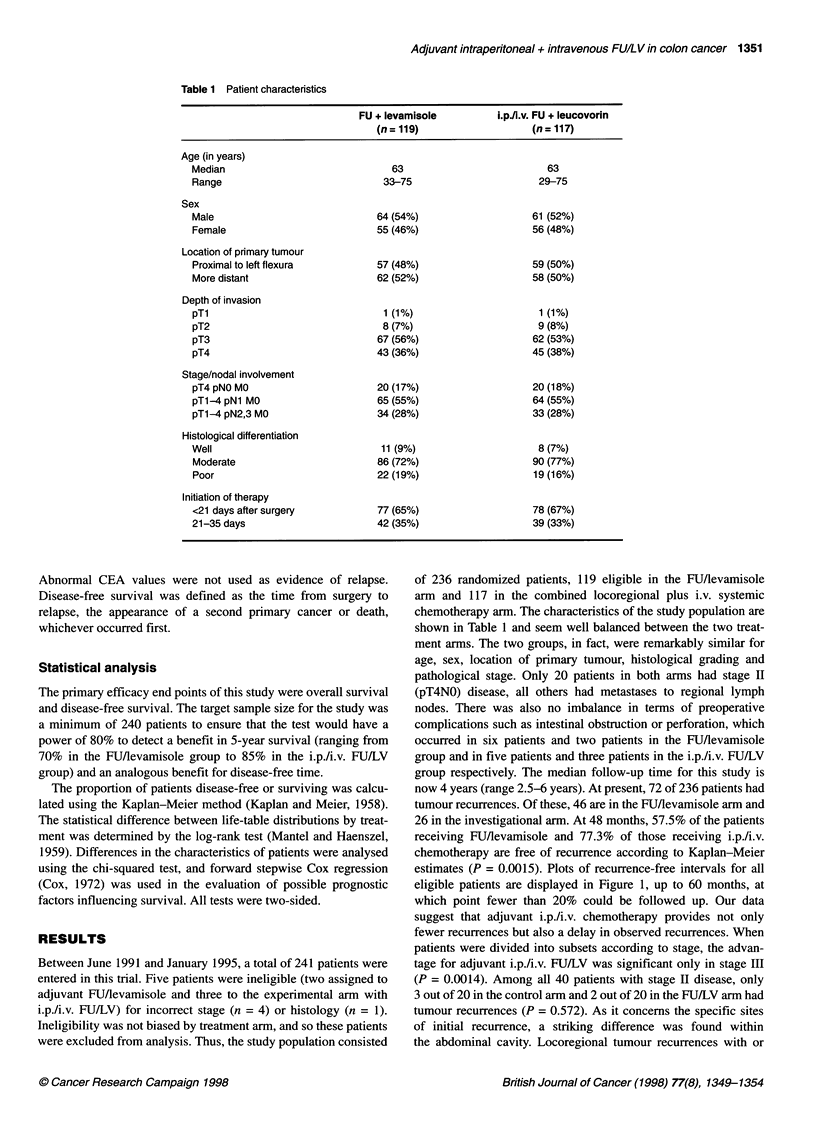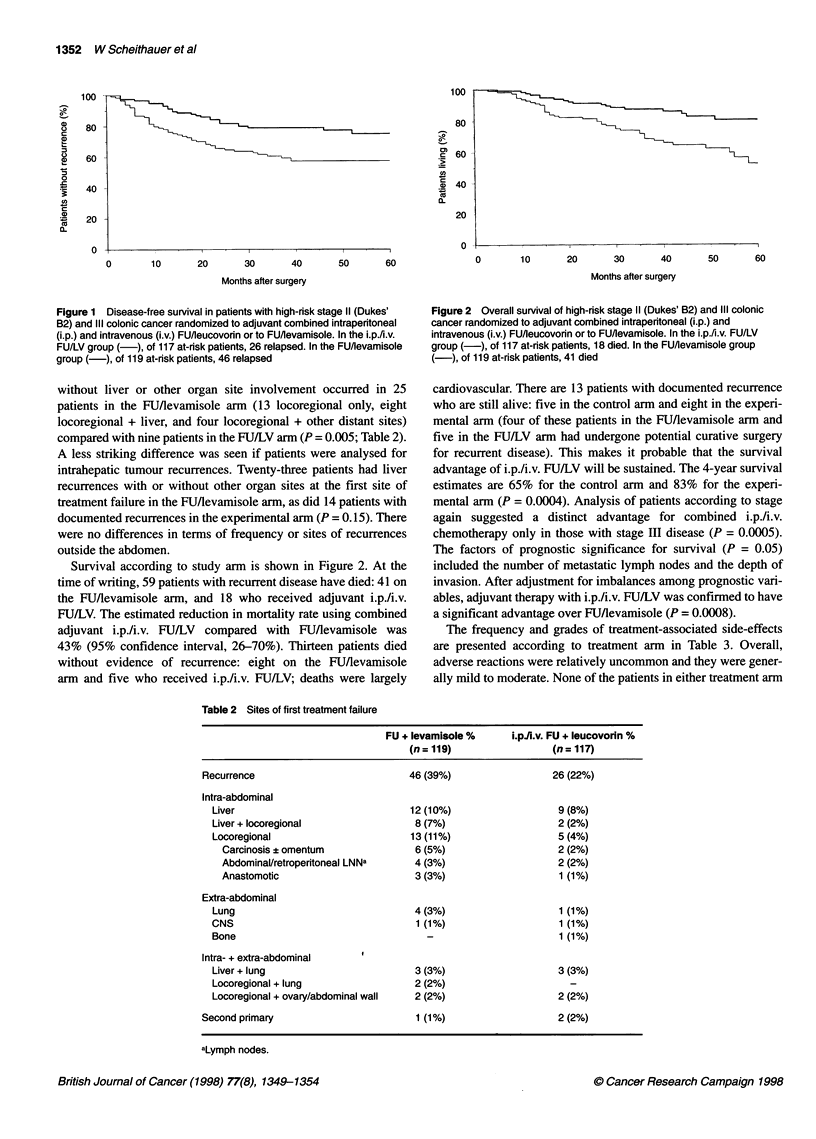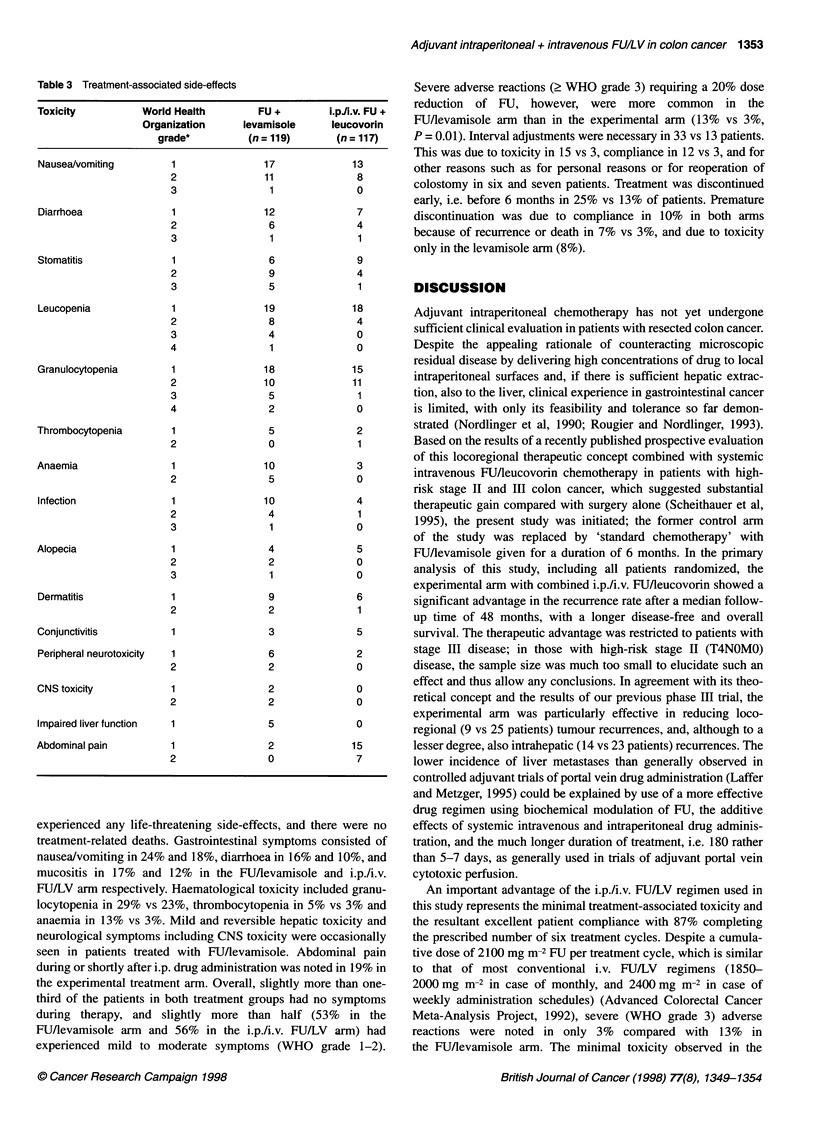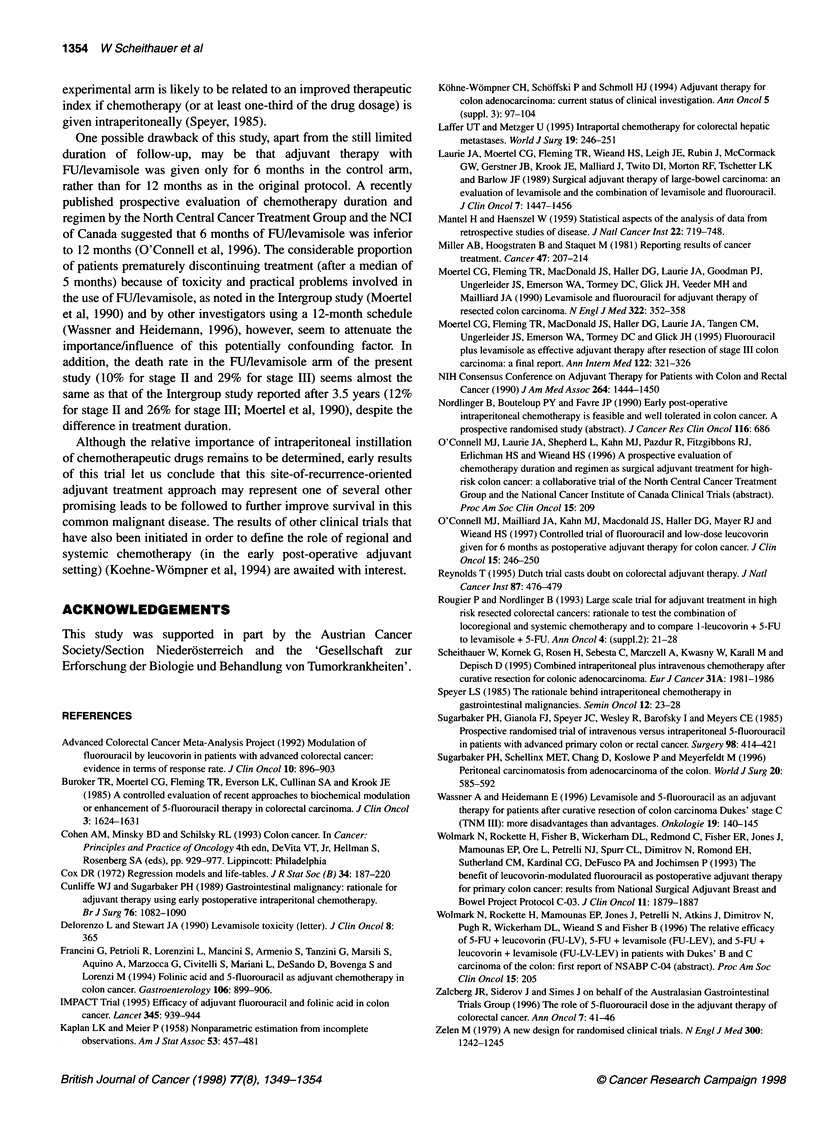# Combined intravenous and intraperitoneal chemotherapy with fluorouracil + leucovorin vs fluorouracil + levamisole for adjuvant therapy of resected colon carcinoma.

**DOI:** 10.1038/bjc.1998.225

**Published:** 1998-04

**Authors:** W. Scheithauer, G. V. Kornek, A. Marczell, J. Karner, G. Salem, R. Greiner, D. Burger, F. StÃ¶ger, J. Ritschel, E. Kovats, H. M. Vischer, B. Schneeweiss, D. Depisch

**Affiliations:** Department of Internal Medicine I, University of Vienna, Austria.

## Abstract

Adjuvant chemotherapy with fluorouracil (FU) and levamisole or FU/leucovorin (LV) has been established as effective adjuvant treatment for patients with stage III colon cancer. Among several other promising treatment strategies in resected colon cancer, intraperitoneal anti-cancer drug administration with its appealing rationale of counteracting microscopic residual disease on peritoneal surfaces and occult metachronous liver metastases by achieving high intraportal drug concentrations has not yet undergone sufficient clinical evaluation. To determine whether a combination of this locoregional therapeutic concept with systemic intravenous administration of FU/LV would yield better results than conventional adjuvant chemoimmunotherapy with FU/levamisole, the present randomized study was initiated. A total of 241 patients with resected stage III or high-risk stage II (T4N0M0) colon cancer were randomly assigned to 'standard therapy' with FU and levamisole, given for a duration of 6 months, or to an investigational arm, consisting of LV 200 mg m(-2) plus FU 350 mg m(-2), both administered intravenously (days 1-4) and intraperitoneally (days 1 and 3) every 4 weeks for a total of six courses. In patients with stage II disease, no significant difference was noted between the two arms after a median follow-up time of 4 years (range 2.5-6 years). Among 196 eligible patients with stage III disease, however, a comparative analysis of the two treatment groups suggested both an improvement in disease-free survival (P = 0.0014) and a survival advantage (P = 0.0005), with an estimated 43% reduction in mortality rate (95% confidence interval 26-70%) in favour of the investigational arm. In agreement with its theoretical rationale, combined intraperitoneal and intravenous FU/LV was particularly effective in reducing locoregional tumour recurrences with or without liver or other organ site involvement (9 vs 25 patients in the FU/levamisole arm; P = 0.005). Treatment-associated side-effects were infrequent and generally mild in both arms, although a lower rate of severe (WHO grade 3) adverse reactions was noted in patients receiving locoregional plus intravenous chemotherapy (3% vs 12%; P = 0.01). The results of this trial suggest that combined intraperitoneal plus systemic intravenous chemotherapy with FU/LV is a promising adjuvant treatment strategy in patients with surgically resected stage III colon carcinoma.


British Journal of Cancer (1998) 77(8), 1349–1354
© 1998 Cancer Research Campaign

### Combined intravenous and intraperitoneal chemotherapy with fluorouracil + leucovorin vs fluorouracil + levamisole for adjuvant therapy of resected colon carcinoma

W Scheithauer[1], GV Kornek[1], A Marczell[2], J Karner[3], G Salem[4], R Greiner[4], D Burger[5], F Stöger[6], J Ritschel[6], E Kovats[7], HM Vischer[8], B Schneeweiss[9] and D Depisch[10]

[1]Department of Internal Medicine I, Division of Oncology, University of Vienna, Waehringer Guertel 18–20, A-1090 Vienna; [2]Department of Surgery, Hanusch-Hospital, Heinrich-Collin-Strasse 30, A-1140 Vienna; [3]Department of Surgery, Kaiser-Franz-Josef Hospital, Kundratstrasse 3, A-1100 Vienna; [4]General Hospital of St Poelten, Propst-Fuehrer-Strasse 4, A-3100 St Poelten; [5]Barmherzige Brueder Hospital, Grosse Mohrengasse 9, A-1021 Vienna; General Hospitals of [6]Tulln, Alter Ziegelweg 50, A-3430 Tulln, [7]Baden, Wimmergasse 19, A-2500 Baden, [8]Gmuend, Conrathstrasse 17, A-3952 Gmuend, [9]Kirchdorf, A-4560 Kirchdorf a.d.Krems, [10]Wr. Neustadt, Corvinusring 3–5, A-2700 Wr.Neustadt, Austria

**Summary** Adjuvant chemotherapy with fluorouracil (FU) and levamisole or FU/leucovorin (LV) has been established as effective adjuvant treatment for patients with stage III colon cancer. Among several other promising treatment strategies in resected colon cancer, intraperitoneal anti-cancer drug administration with its appealing rationale of counteracting microscopic residual disease on peritoneal surfaces and occult metachronous liver metastases by achieving high intraportal drug concentrations has not yet undergone sufficient clinical evaluation. To determine whether a combination of this locoregional therapeutic concept with systemic intravenous administration of FU/LV would yield better results than conventional adjuvant chemoimmunotherapy with FU/levamisole, the present randomized study was initiated. A total of 241 patients with resected stage III or high-risk stage II (T4N0M0) colon cancer were randomly assigned to 'standard therapy' with FU and levamisole, given for a duration of 6 months, or to an investigational arm, consisting of LV 200 mg $m^{-2}$ plus FU 350 mg $m^{-2}$, both administered intravenously (days 1–4) and intraperitoneally (days 1 and 3) every 4 weeks for a total of six courses. In patients with stage II disease, no significant difference was noted between the two arms after a median follow-up time of 4 years (range 2.5–6 years). Among 196 eligible patients with stage III disease, however, a comparative analysis of the two treatment groups suggested both an improvement in disease-free survival ($P = 0.0014$) and a survival advantage ($P = 0.0005$), with an estimated 43% reduction in mortality rate (95% confidence interval 26–70%) in favour of the investigational arm. In agreement with its theoretical rationale, combined intraperitoneal and intravenous FU/LV was particularly effective in reducing locoregional tumour recurrences with or without liver or other organ site involvement (9 vs 25 patients in the FU/levamisole arm; $P = 0.005$). Treatment-associated side-effects were infrequent and generally mild in both arms, although a lower rate of severe (WHO grade 3) adverse reactions was noted in patients receiving locoregional plus intravenous chemotherapy (3% vs 12%; $P = 0.01$). The results of this trial suggest that combined intraperitoneal plus systemic intravenous chemotherapy with FU/LV is a promising adjuvant treatment strategy in patients with surgically resected stage III colon carcinoma.

**Keywords**: colon carcinoma; adjuvant therapy; fluorouracil; levamisole; leucovorin; intraperitoneal chemotherapy

Adenocarcinoma of the colon is one of the most common internal malignancies, affecting about 1 person in 20 in the Western world (Cohen et al, 1993). Although most patients present with surgically resectable disease, almost half die of the cancer because of occult micrometastases already present at the time of initial diagnosis. In recent years, however, there have been some advances. In fact, it is now over 5 years since the publication of two randomized trials showing that fluorouracil (FU) and levamisole reduced the relapse rate by 40% and mortality by a third in patients with node-positive colon cancer (Laurie et al, 1989; Moertel et al, 1990). The final report of these data was recently published, showing 168 deaths in untreated control patients vs only 121 in those who received adjuvant treatment ($P > 0.005$) (Moertel et al, 1995). Despite these data and recommendation of this regimen as standard therapy for Dukes' C patients since the consensus conference of the National Institute of Health (NIH, 1990), many (European) surgeons and oncologists remained sceptical about its benefits (Reynolds, 1995; Wassner and Heidemann, 1996; Zalcberg et al, 1996). The adjuvant FU/levamisole regimen has been defined empirically; the exact therapeutic role and anti-tumour mechanism of action of the antihelmintic drug levamisole remains uncertain, treatment is not without toxicity (Delorenzo and Stewart, 1990; Wassner and Heidemann, 1996), compliance with weekly drug administration was noted to be poor (Delorenzo and Stewart, 1990; Moertel et al, 1990), and in patients with metastatic disease this combination does not show any advantage over FU alone (Buroker et al, 1985).

The most active chemotherapeutic regimen in patients with advanced colorectal cancer represents the biochemical modulation of FU by leucovorin (LV). A number of prospective randomized

*Received 8 July 1997*
*Revised 25 September 1997*
*Accepted 7 October 1997*

*Correspondence to:* W Scheithauer, Division of Oncology, Department of Internal Medicine I, University of Vienna, Währinger Guertel 18–20, A-1090 Vienna, Austria trials have demonstrated its superior response activity compared with FU monotherapy (Advanced Colorectal Cancer Meta-Analysis Project, 1992). There is also accumulating evidence that FU/LV is very effective in the adjuvant treatment setting (Wolmark et al, 1993; Francini et al, 1994; IMPACT Trial, 1995; O'Connell et al, 1997), and recent data suggest that it even may be superior in terms of survival and disease-free survival compared with FU/levamisole (Wolmark et al, 1996).

In the present study, we have randomized patients between adjuvant FU plus LV administered both by systemic intravenous (i.v.) infusion and intraperitoneally (i.p.) and 'standard adjuvant therapy' with FU plus levamisole. The rationale for the combined i.p. + i.v. mode of drug administration was to counteract tumour dissemination via haematogenous/lymphogenous spread as well as perioperative implantation of tumour cells in the resection site and in peritoneal surfaces, which represent common sites of colonic cancer recurrence (Sugarbaker et al, 1985; 1996; Cunliffe and Sugarbaker, 1989). Pharmacokinetic studies with i.p. FU have demonstrated that tumoricidal doses of the drug are present in the abdominal cavity for at least 8 h after instillation (Cunliffe and Sugarbaker, 1989). In addition, up to ten times the level of drug is seen in the portal vein than is noted in the peripheral blood (Sugarbaker et al, 1985; Rougier and Nordlinger, 1993). Thus, i.p./i.v. administration of FU/LV could not only protect peritoneal surfaces, but also counteract occult metachronous liver metastases by achieving high intraportal/intrahepatic drug concentrations. Feasibility, tolerance and the therapeutic potential of this theoretically appealing adjuvant treatment approach has been demonstrated in a previous study in patients with stage III and high-risk stage II colon cancer: after almost 5 years of follow-up, a significant difference in recurrence-free survival and an overall survival advantage of 78% vs 63% was noted in patients given i.p. + i.v. FU/LV compared with an untreated control (Scheithauer et al, 1995).

### PATIENTS AND METHODS

### Patient selection

To be included in the study, patients were required to have undergone curative en bloc resection of an adenocarcinoma of the colon without gross or microscopic evidence of residual disease. Patients had to have histopathological diagnosis of stage II with invasion extending at least to the serosa or pericolonic fat (Dukes' B2) or stage III (Dukes' C) disease. At least ten regional lymph nodes had to be examined. Additional eligibility criteria included age 75 years or younger, a World Health Organization (WHO) performance status less than 2, normal bone marrow (leucocytes > 4 000 $\mu l^{-1}$, thrombocytes > 100 000 $\mu l^{-1}$), liver (bilirubin <1.5 mg $dl^{-1}$; transaminase level less than two times the upper limit of normal), and renal functions (serum creatinine <1.5 mg $dl^{-1}$). Patients were excluded if they had rectal cancer (defined using this protocol as any lesion that required the opening of the pelvic peritoneum to define the distal extent of the tumour), any other cancer except for superficial skin carcinoma or in situ carcinoma of the cervix, or any other severe concomitant disease that would preclude a normal life expectancy of at least 5 years. No other adjuvant therapy was allowed. Eligibility was determined by careful review of study forms, operative and pathology reports. Entry into the study was allowed no earlier than 1 week and no later than 5 weeks after surgery.

### Randomization and treatment

Before randomization, each patient was physically examined, had routine haematological testing and blood chemistry including carcinoembryonic antigen (CEA), a chest radiogram and abdominal sonography and/or computerized tomography (CT) scan. Informed consent according to institutional regulations was obtained from all patients. Eligible patients were registered by phone at the central statistical office of the University of Vienna. They were stratified according to participating centre, tumour stage (II vs III with < 4 or ≥ 4 lymph nodes involved), extent of invasion (into or through the serosa vs into adjacent organs) and interval since surgery (< 21 vs 21–35 days).

They were then randomly assigned to adjuvant therapy with FU/levamisole or i.v./i.p. FU/LV according to the Zelen method (Zelen, 1979). Dosages for FU and levamisole were the same as those used in the National Intergroup Study (Moertel et al, 1990), although patients were treated only for a total of 6 months. Levamisole 50 mg was given orally every 8 h for a period of 3 days, repeated every 2 weeks. FU 450 mg $m^{-2}$ was given by rapid i.v. injection daily for 5 consecutive days; 28 days after the start of the first course, FU was continued weekly. In the experimental treatment arm, patients received LV 200 mg $m^{-2}$ and FU 350 mg $m^{-2}$ both administered by i.v. bolus injection daily for 4 consecutive days. On days 1 and 3 of each treatment cycle, LV and FU, each diluted in 500 ml of saline, were also given intraperitoneally in the same sequence and the same dosage. Intraperitoneal drug administration was usually performed with a peripheral venous catheter under local anaesthesia, although 14 patients underwent surgical placement of an i.p. catheter attached to a subcutaneous reservoir permitting transdermal access to the catheter. Each 4-day course of i.p./i.v. adjuvant therapy was repeated every 4 weeks for a total of six cycles. This particular i.p./i.v. treatment schedule was based on a small pilot study and our previous phase III trial (Scheithauer et al, 1995), indicating potential therapeutic benefit and minimal toxicity despite use of a cumulative dose of the drugs comparable with that of most conventional i.v. FU/LV regimens used in the adjuvant/palliative treatment setting.

Toxicity was assessed according to WHO standard criteria (Miller et al, 1981). If stomatitis, diarrhoea, or leucopenia developed, chemotherapy was deferred until the side-effects subsided. If toxicity persisted for more than 2 weeks, or if it exceeded WHO grade 2, the dose of FU was reduced by 20% in both treatment arms. Complete blood cell counts were obtained every 2 weeks during therapy, and serum electrolytes, liver and kidney function parameters were repeated every 4 weeks.

### Follow-up

All randomized patients were followed up for recurrence and survival every 3 months during the first 2 years, thereafter every 6 months for a total of 5 years. Examinations consisted of an interim history taking, physical examination and blood chemistry, including CEA. Chest radiography, abdominal sonography and/or CT scans and examination of the entire colorectum (barium enema or endoscopy) was performed every 6 months and annually after the second year. A documented histological diagnosis by percutaneous or colonoscopic biopsy or reoperation was required to confirm recurrence of tumour, except in cases of lung or liver metastases with unequivocal radiographic or scan changes.

 © Cancer Research Campaign 1998

**Table 1** Patient characteristics

| | FU + levamisole ($n$ = 119) | i.p./i.v. FU + leucovorin ($n$ = 117) |
|---|---|---|
| Age (in years) | | |
| Median | 63 | 63 |
| Range | 33–75 | 29–75 |
| Sex | | |
| Male | 64 (54%) | 61 (52%) |
| Female | 55 (46%) | 56 (48%) |
| Location of primary tumour | | |
| Proximal to left flexura | 57 (48%) | 59 (50%) |
| More distant | 62 (52%) | 58 (50%) |
| Depth of invasion | | |
| pT1 | 1 (1%) | 1 (1%) |
| pT2 | 8 (7%) | 9 (8%) |
| pT3 | 67 (56%) | 62 (53%) |
| pT4 | 43 (36%) | 45 (38%) |
| Stage/nodal involvement | | |
| pT4 pN0 M0 | 20 (17%) | 20 (18%) |
| pT1–4 pN1 M0 | 65 (55%) | 64 (55%) |
| pT1–4 pN2,3 M0 | 34 (28%) | 33 (28%) |
| Histological differentiation | | |
| Well | 11 (9%) | 8 (7%) |
| Moderate | 86 (72%) | 90 (77%) |
| Poor | 22 (19%) | 19 (16%) |
| Initiation of therapy | | |
| <21 days after surgery | 77 (65%) | 78 (67%) |
| 21–35 days | 42 (35%) | 39 (33%) |

Abnormal CEA values were not used as evidence of relapse. Disease-free survival was defined as the time from surgery to relapse, the appearance of a second primary cancer or death, whichever occurred first.

### Statistical analysis

The primary efficacy end points of this study were overall survival and disease-free survival. The target sample size for the study was a minimum of 240 patients to ensure that the test would have a power of 80% to detect a benefit in 5-year survival (ranging from 70% in the FU/levamisole group to 85% in the i.p./i.v. FU/LV group) and an analogous benefit for disease-free time.

The proportion of patients disease-free or surviving was calculated using the Kaplan–Meier method (Kaplan and Meier, 1958). The statistical difference between life-table distributions by treatment was determined by the log-rank test (Mantel and Haenszel, 1959). Differences in the characteristics of patients were analysed using the chi-squared test, and forward stepwise Cox regression (Cox, 1972) was used in the evaluation of possible prognostic factors influencing survival. All tests were two-sided.

### RESULTS

Between June 1991 and January 1995, a total of 241 patients were entered in this trial. Five patients were ineligible (two assigned to adjuvant FU/levamisole and three to the experimental arm with i.p./i.v. FU/LV) for incorrect stage ($n$ = 4) or histology ($n$ = 1). Ineligibility was not biased by treatment arm, and so these patients were excluded from analysis. Thus, the study population consisted of 236 randomized patients, 119 eligible in the FU/levamisole arm and 117 in the combined locoregional plus i.v. systemic chemotherapy arm. The characteristics of the study population are shown in Table 1 and seem well balanced between the two treatment arms. The two groups, in fact, were remarkably similar for age, sex, location of primary tumour, histological grading and pathological stage. Only 20 patients in both arms had stage II (pT4N0) disease, all others had metastases to regional lymph nodes. There was also no imbalance in terms of preoperative complications such as intestinal obstruction or perforation, which occurred in six patients and two patients in the FU/levamisole group and in five patients and three patients in the i.p./i.v. FU/LV group respectively. The median follow-up time for this study is now 4 years (range 2.5–6 years). At present, 72 of 236 patients had tumour recurrences. Of these, 46 are in the FU/levamisole arm and 26 in the investigational arm. At 48 months, 57.5% of the patients receiving FU/levamisole and 77.3% of those receiving i.p./i.v. chemotherapy are free of recurrence according to Kaplan–Meier estimates ($P$ = 0.0015). Plots of recurrence-free intervals for all eligible patients are displayed in Figure 1, up to 60 months, at which point fewer than 20% could be followed up. Our data suggest that adjuvant i.p./i.v. chemotherapy provides not only fewer recurrences but also a delay in observed recurrences. When patients were divided into subsets according to stage, the advantage for adjuvant i.p./i.v. FU/LV was significant only in stage III ($P$ = 0.0014). Among all 40 patients with stage II disease, only 3 out of 20 in the control arm and 2 out of 20 in the FU/LV arm had tumour recurrences ($P$ = 0.572). As it concerns the specific sites of initial recurrence, a striking difference was found within the abdominal cavity. Locoregional tumour recurrences with or

© Cancer Research Campaign 1998

**Figure 1** Disease-free survival in patients with high-risk stage II (Dukes' B2) and III colonic cancer randomized to adjuvant combined intraperitoneal (i.p.) and intravenous (i.v.) FU/leucovorin or to FU/levamisole. In the i.p./i.v. FU/LV group (——), of 117 at-risk patients, 26 relapsed. In the FU/levamisole group (——), of 119 at-risk patients, 46 relapsed

**Figure 2** Overall survival of high-risk stage II (Dukes' B2) and III colonic cancer randomized to adjuvant combined intraperitoneal (i.p.) and intravenous (i.v.) FU/leucovorin or to FU/levamisole. In the i.p./i.v. FU/LV group (——), of 117 at-risk patients, 18 died. In the FU/levamisole group (——), of 119 at-risk patients, 41 died without liver or other organ site involvement occurred in 25 patients in the FU/levamisole arm (13 locoregional only, eight locoregional + liver, and four locoregional + other distant sites) compared with nine patients in the FU/LV arm ($P = 0.005$; Table 2). A less striking difference was seen if patients were analysed for intrahepatic tumour recurrences. Twenty-three patients had liver recurrences with or without other organ sites at the first site of treatment failure in the FU/levamisole arm, as did 14 patients with documented recurrences in the experimental arm ($P = 0.15$). There were no differences in terms of frequency or sites of recurrences outside the abdomen.

Survival according to study arm is shown in Figure 2. At the time of writing, 59 patients with recurrent disease have died: 41 on the FU/levamisole arm, and 18 who received adjuvant i.p./i.v. FU/LV. The estimated reduction in mortality rate using combined adjuvant i.p./i.v. FU/LV compared with FU/levamisole was 43% (95% confidence interval, 26–70%). Thirteen patients died without evidence of recurrence: eight on the FU/levamisole arm and five who received i.p./i.v. FU/LV; deaths were largely cardiovascular. There are 13 patients with documented recurrence who are still alive: five in the control arm and eight in the experimental arm (four of these patients in the FU/levamisole arm and five in the FU/LV arm had undergone potential curative surgery for recurrent disease). This makes it probable that the survival advantage of i.p./i.v. FU/LV will be sustained. The 4-year survival estimates are 65% for the control arm and 83% for the experimental arm ($P = 0.0004$). Analysis of patients according to stage again suggested a distinct advantage for combined i.p./i.v. chemotherapy only in those with stage III disease ($P = 0.0005$). The factors of prognostic significance for survival ($P = 0.05$) included the number of metastatic lymph nodes and the depth of invasion. After adjustment for imbalances among prognostic variables, adjuvant therapy with i.p./i.v. FU/LV was confirmed to have a significant advantage over FU/levamisole ($P = 0.0008$).

The frequency and grades of treatment-associated side-effects are presented according to treatment arm in Table 3. Overall, adverse reactions were relatively uncommon and they were generally mild to moderate. None of the patients in either treatment arm

**Table 2** Sites of first treatment failure

| | FU + levamisole % (*n* = 119) | i.p./i.v. FU + leucovorin % (*n* = 117) |
|---|---|---|
| Recurrence | 46 (39%) | 26 (22%) |
| Intra-abdominal | | |
| Liver | 12 (10%) | 9 (8%) |
| Liver + locoregional | 8 (7%) | 2 (2%) |
| Locoregional | 13 (11%) | 5 (4%) |
| Carcinosis ± omentum | 6 (5%) | 2 (2%) |
| Abdominal/retroperitoneal LNN[a] | 4 (3%) | 2 (2%) |
| Anastomotic | 3 (3%) | 1 (1%) |
| Extra-abdominal | | |
| Lung | 4 (3%) | 1 (1%) |
| CNS | 1 (1%) | 1 (1%) |
| Bone | – | 1 (1%) |
| Intra- + extra-abdominal | | |
| Liver + lung | 3 (3%) | 3 (3%) |
| Locoregional + lung | 2 (2%) | – |
| Locoregional + ovary/abdominal wall | 2 (2%) | 2 (2%) |
| Second primary | 1 (1%) | 2 (2%) |

[a]Lymph nodes.

 © Cancer Research Campaign 1998

**Table 3** Treatment-associated side-effects

| Toxicity | World Health Organization grade* | FU + levamisole ($n$ = 119) | i.p./i.v. FU + leucovorin ($n$ = 117) |
|---|---|---|---|
| Nausea/vomiting | 1 | 17 | 13 |
| | 2 | 11 | 8 |
| | 3 | 1 | 0 |
| Diarrhoea | 1 | 12 | 7 |
| | 2 | 6 | 4 |
| | 3 | 1 | 1 |
| Stomatitis | 1 | 6 | 9 |
| | 2 | 9 | 4 |
| | 3 | 5 | 1 |
| Leucopenia | 1 | 19 | 18 |
| | 2 | 8 | 4 |
| | 3 | 4 | 0 |
| | 4 | 1 | 0 |
| Granulocytopenia | 1 | 18 | 15 |
| | 2 | 10 | 11 |
| | 3 | 5 | 1 |
| | 4 | 2 | 0 |
| Thrombocytopenia | 1 | 5 | 2 |
| | 2 | 0 | 1 |
| Anaemia | 1 | 10 | 3 |
| | 2 | 5 | 0 |
| Infection | 1 | 10 | 4 |
| | 2 | 4 | 1 |
| | 3 | 1 | 0 |
| Alopecia | 1 | 4 | 5 |
| | 2 | 2 | 0 |
| | 3 | 1 | 0 |
| Dermatitis | 1 | 9 | 6 |
| | 2 | 2 | 1 |
| Conjunctivitis | 1 | 3 | 5 |
| Peripheral neurotoxicity | 1 | 6 | 2 |
| | 2 | 2 | 0 |
| CNS toxicity | 1 | 2 | 0 |
| | 2 | 2 | 0 |
| Impaired liver function | 1 | 5 | 0 |
| Abdominal pain | 1 | 2 | 15 |
| | 2 | 0 | 7 |

experienced any life-threatening side-effects, and there were no treatment-related deaths. Gastrointestinal symptoms consisted of nausea/vomiting in 24% and 18%, diarrhoea in 16% and 10%, and mucositis in 17% and 12% in the FU/levamisole and i.p./i.v. FU/LV arm respectively. Haematological toxicity included granulocytopenia in 29% vs 23%, thrombocytopenia in 5% vs 3% and anaemia in 13% vs 3%. Mild and reversible hepatic toxicity and neurological symptoms including CNS toxicity were occasionally seen in patients treated with FU/levamisole. Abdominal pain during or shortly after i.p. drug administration was noted in 19% in the experimental treatment arm. Overall, slightly more than one-third of the patients in both treatment groups had no symptoms during therapy, and slightly more than half (53% in the FU/levamisole arm and 56% in the i.p./i.v. FU/LV arm) had experienced mild to moderate symptoms (WHO grade 1–2). Severe adverse reactions (≥ WHO grade 3) requiring a 20% dose reduction of FU, however, were more common in the FU/levamisole arm than in the experimental arm (13% vs 3%, $P = 0.01$). Interval adjustments were necessary in 33 vs 13 patients. This was due to toxicity in 15 vs 3, compliance in 12 vs 3, and for other reasons such as for personal reasons or for reoperation of colostomy in six and seven patients. Treatment was discontinued early, i.e. before 6 months in 25% vs 13% of patients. Premature discontinuation was due to compliance in 10% in both arms because of recurrence or death in 7% vs 3%, and due to toxicity only in the levamisole arm (8%).

### DISCUSSION

Adjuvant intraperitoneal chemotherapy has not yet undergone sufficient clinical evaluation in patients with resected colon cancer. Despite the appealing rationale of counteracting microscopic residual disease by delivering high concentrations of drug to local intraperitoneal surfaces and, if there is sufficient hepatic extraction, also to the liver, clinical experience in gastrointestinal cancer is limited, with only its feasibility and tolerance so far demonstrated (Nordlinger et al, 1990; Rougier and Nordlinger, 1993). Based on the results of a recently published prospective evaluation of this locoregional therapeutic concept combined with systemic intravenous FU/leucovorin chemotherapy in patients with high-risk stage II and III colon cancer, which suggested substantial therapeutic gain compared with surgery alone (Scheithauer et al, 1995), the present study was initiated; the former control arm of the study was replaced by 'standard chemotherapy' with FU/levamisole given for a duration of 6 months. In the primary analysis of this study, including all patients randomized, the experimental arm with combined i.p./i.v. FU/leucovorin showed a significant advantage in the recurrence rate after a median follow-up time of 48 months, with a longer disease-free and overall survival. The therapeutic advantage was restricted to patients with stage III disease; in those with high-risk stage II (T4N0M0) disease, the sample size was much too small to elucidate such an effect and thus allow any conclusions. In agreement with its theoretical concept and the results of our previous phase III trial, the experimental arm was particularly effective in reducing locoregional (9 vs 25 patients) tumour recurrences, and, although to a lesser degree, also intrahepatic (14 vs 23 patients) recurrences. The lower incidence of liver metastases than generally observed in controlled adjuvant trials of portal vein drug administration (Laffer and Metzger, 1995) could be explained by use of a more effective drug regimen using biochemical modulation of FU, the additive effects of systemic intravenous and intraperitoneal drug administration, and the much longer duration of treatment, i.e. 180 rather than 5–7 days, as generally used in trials of adjuvant portal vein cytotoxic perfusion.

An important advantage of the i.p./i.v. FU/LV regimen used in this study represents the minimal treatment-associated toxicity and the resultant excellent patient compliance with 87% completing the prescribed number of six treatment cycles. Despite a cumulative dose of 2100 mg $m^{-2}$ FU per treatment cycle, which is similar to that of most conventional i.v. FU/LV regimens (1850–2000 mg $m^{-2}$ in case of monthly, and 2400 mg $m^{-2}$ in case of weekly administration schedules) (Advanced Colorectal Cancer Meta-Analysis Project, 1992), severe (WHO grade 3) adverse reactions were noted in only 3% compared with 13% in the FU/levamisole arm. The minimal toxicity observed in the experimental arm is likely to be related to an improved therapeutic index if chemotherapy (or at least one-third of the drug dosage) is given intraperitoneally (Speyer, 1985).

One possible drawback of this study, apart from the still limited duration of follow-up, may be that adjuvant therapy with FU/levamisole was given only for 6 months in the control arm, rather than for 12 months as in the original protocol. A recently published prospective evaluation of chemotherapy duration and regimen by the North Central Cancer Treatment Group and the NCI of Canada suggested that 6 months of FU/levamisole was inferior to 12 months (O'Connell et al, 1996). The considerable proportion of patients prematurely discontinuing treatment (after a median of 5 months) because of toxicity and practical problems involved in the use of FU/levamisole, as noted in the Intergroup study (Moertel et al, 1990) and by other investigators using a 12-month schedule (Wassner and Heidemann, 1996), however, seem to attenuate the importance/influence of this potentially confounding factor. In addition, the death rate in the FU/levamisole arm of the present study (10% for stage II and 29% for stage III) seems almost the same as that of the Intergroup study reported after 3.5 years (12% for stage II and 26% for stage III; Moertel et al, 1990), despite the difference in treatment duration.

Although the relative importance of intraperitoneal instillation of chemotherapeutic drugs remains to be determined, early results of this trial let us conclude that this site-of-recurrence-oriented adjuvant treatment approach may represent one of several other promising leads to be followed to further improve survival in this common malignant disease. The results of other clinical trials that have also been initiated in order to define the role of regional and systemic chemotherapy (in the early post-operative adjuvant setting) (Koehne-Wömpner et al, 1994) are awaited with interest.

### ACKNOWLEDGEMENTS

This study was supported in part by the Austrian Cancer Society/Section Niederösterreich and the 'Gesellschaft zur Erforschung der Biologie und Behandlung von Tumorkrankheiten'.

### REFERENCES

Advanced Colorectal Cancer Meta-Analysis Project (1992) Modulation of fluorouracil by leucovorin in patients with advanced colorectal cancer: evidence in terms of response rate. *J Clin Oncol* **10**: 896–903

Buroker TR, Moertel CG, Fleming TR, Everson LK, Cullinan SA and Krook JE (1985) A controlled evaluation of recent approaches to biochemical modulation or enhancement of 5-fluorouracil therapy in colorectal carcinoma. *J Clin Oncol* **3**: 1624–1631

Cohen AM, Minsky BD and Schilsky RL (1993) Colon cancer. In *Cancer: Principles and Practice of Oncology* 4th edn, DeVita VT, Jr, Hellman S, Rosenberg SA (eds), pp. 929–977. Lippincott: Philadelphia

Cox DR (1972) Regression models and life-tables. *J R Stat Soc (B)* **34**: 187–220

Cunliffe WJ and Sugarbaker PH (1989) Gastrointestinal malignancy: rationale for adjuvant therapy using early postoperative intraperitonal chemotherapy. *Br J Surg* **76**: 1082–1090

Delorenzo L and Stewart JA (1990) Levamisole toxicity (letter). *J Clin Oncol* **8**: 365

Francini G, Petrioli R, Lorenzini L, Mancini S, Armenio S, Tanzini G, Marsili S, Aquino A, Marzocca G, Civitelli S, Mariani L, DeSando D, Bovenga S and Lorenzi M (1994) Folinic acid and 5-fluorouracil as adjuvant chemotherapy in colon cancer. *Gastroenterology* **106**: 899–906.

IMPACT Trial (1995) Efficacy of adjuvant fluorouracil and folinic acid in colon cancer. *Lancet* **345**: 939–944

Kaplan LK and Meier P (1958) Nonparametric estimation from incomplete observations. *Am J Stat Assoc* **53**: 457–481

Köhne-Wömpner CH, Schöffski P and Schmoll HJ (1994) Adjuvant therapy for colon adenocarcinoma: current status of clinical investigation. *Ann Oncol* **5** (suppl. 3): 97–104

Laffer UT and Metzger U (1995) Intraportal chemotherapy for colorectal hepatic metastases. *World J Surg* **19**: 246–251

Laurie JA, Moertel CG, Fleming TR, Wieand HS, Leigh JE, Rubin J, McCormack GW, Gerstner JB, Krook JE, Malliard J, Twito DI, Morton RF, Tschetter LK and Barlow JF (1989) Surgical adjuvant therapy of large-bowel carcinoma: an evaluation of levamisole and the combination of levamisole and fluorouracil. *J Clin Oncol* **7**: 1447–1456

Mantel H and Haenszel W (1959) Statistical aspects of the analysis of data from retrospective studies of disease. *J Natl Cancer Inst* **22**: 719–748.

Miller AB, Hoogstraten B and Staquet M (1981) Reporting results of cancer treatment. *Cancer* **47**: 207–214

Moertel CG, Fleming TR, MacDonald JS, Haller DG, Laurie JA, Goodman PJ, Ungerleider JS, Emerson WA, Tormey DC, Glick JH, Veeder MH and Mailliard JA (1990) Levamisole and fluorouracil for adjuvant therapy of resected colon carcinoma. *N Engl J Med* **322**: 352–358

Moertel CG, Fleming TR, MacDonald JS, Haller DG, Laurie JA, Tangen CM, Ungerleider JS, Emerson WA, Tormey DC and Glick JH (1995) Fluorouracil plus levamisole as effective adjuvant therapy after resection of stage III colon carcinoma: a final report. *Ann Intern Med* **122**: 321–326

NIH Consensus Conference on Adjuvant Therapy for Patients with Colon and Rectal Cancer (1990) *J Am Med Assoc* **264**: 1444–1450

Nordlinger B, Bouteloup PY and Favre JP (1990) Early post-operative intraperitoneal chemotherapy is feasible and well tolerated in colon cancer. A prospective randomised study (abstract). *J Cancer Res Clin Oncol* **116**: 686

O'Connell MJ, Laurie JA, Shepherd L, Kahn MJ, Pazdur R, Fitzgibbons RJ, Erlichman HS and Wieand HS (1996) A prospective evaluation of chemotherapy duration and regimen as surgical adjuvant treatment for high-risk colon cancer: a collaborative trial of the North Central Cancer Treatment Group and the National Cancer Institute of Canada Clinical Trials (abstract). *Proc Am Soc Clin Oncol* **15**: 209

O'Connell MJ, Mailliard JA, Kahn MJ, Macdonald JS, Haller DG, Mayer RJ and Wieand HS (1997) Controlled trial of fluorouracil and low-dose leucovorin given for 6 months as postoperative adjuvant therapy for colon cancer. *J Clin Oncol* **15**: 246–250

Reynolds T (1995) Dutch trial casts doubt on colorectal adjuvant therapy. *J Natl Cancer Inst* **87**: 476–479

Rougier P and Nordlinger B (1993) Large scale trial for adjuvant treatment in high risk resected colorectal cancers: rationale to test the combination of locoregional and systemic chemotherapy and to compare 1-leucovorin + 5-FU to levamisole + 5-FU. *Ann Oncol* **4**: (suppl.2): 21–28

Scheithauer W, Kornek G, Rosen H, Sebesta C, Marczell A, Kwasny W, Karall M and Depisch D (1995) Combined intraperitoneal plus intravenous chemotherapy after curative resection for colonic adenocarcinoma. *Eur J Cancer* **31A**: 1981–1986

Speyer LS (1985) The rationale behind intraperitoneal chemotherapy in gastrointestinal malignancies. *Semin Oncol* **12**: 23–28

Sugarbaker PH, Gianola FJ, Speyer JC, Wesley R, Barofsky I and Meyers CE (1985) Prospective randomised trial of intravenous versus intraperitoneal 5-fluorouracil in patients with advanced primary colon or rectal cancer. *Surgery* **98**: 414–421

Sugarbaker PH, Schellinx MET, Chang D, Koslowe P and Meyerfeldt M (1996) Peritoneal carcinomatosis from adenocarcinoma of the colon. *World J Surg* **20**: 585–592

Wassner A and Heidemann E (1996) Levamisole and 5-fluorouracil as an adjuvant therapy for patients after curative resection of colon carcinoma Dukes' stage C (TNM III): more disadvantages than advantages. *Onkologie* **19**: 140–145

Wolmark N, Rockette H, Fisher B, Wickerham DL, Redmond C, Fisher ER, Jones J, Mamounas EP, Ore L, Petrelli NJ, Spurr CL, Dimitrov N, Romond EH, Sutherland CM, Kardinal CG, DeFusco PA and Jochimsen P (1993) The benefit of leucovorin-modulated fluorouracil as postoperative adjuvant therapy for primary colon cancer: results from National Surgical Adjuvant Breast and Bowel Project Protocol C-03. *J Clin Oncol* **11**: 1879–1887

Wolmark N, Rockette H, Mamounas EP, Jones J, Petrelli N, Atkins J, Dimitrov N, Pugh R, Wickerham DL, Wieand S and Fisher B (1996) The relative efficacy of 5-FU + leucovorin (FU-LV), 5-FU + levamisole (FU-LEV), and 5-FU + leucovorin + levamisole (FU-LV-LEV) in patients with Dukes' B and C carcinoma of the colon: first report of NSABP C-04 (abstract). *Proc Am Soc Clin Oncol* **15**: 205

Zalcberg JR, Siderov J and Simes J on behalf of the Australasian Gastrointestinal Trials Group (1996) The role of 5-fluorouracil dose in the adjuvant therapy of colorectal cancer. *Ann Oncol* **7**: 41–46

Zelen M (1979) A new design for randomised clinical trials. *N Engl J Med* **300**: 1242–1245

 © Cancer Research Campaign 1998